# LGBTQIA health in medical education: a national survey of Australian medical students

**DOI:** 10.1186/s12909-024-05099-6

**Published:** 2024-07-07

**Authors:** Sophia Nicolades Wynn, Pravik Solanki, Jayde Millington, Anthony Copeland, Jessie Lu, Ruth McNair, Asiel Adan Sanchez

**Affiliations:** 1 The Australian Medical Students Association, Sydney, New South Wales Australia; 2 The University of Queensland, Brisbane, QLD Australia; 3 Monash University, Clayton, Victoria Australia; 4 The University of Western Australia, Crawley, WA Australia; 5 Department of General Practice, The University of Melbourne, Parkville, Victoria Australia; 6 School of Medicine, The University of Melbourne, Parkville, Victoria Australia

**Keywords:** Health education, Medical curriculum, Queer, LGBTQIA, Sexuality, Gender

## Abstract

**Purpose:**

Lesbian, gay, bisexual, transgender, queer, intersex and asexual (LGBTQIA) individuals experience poorer health outcomes than other individuals. Insufficient LGBTQIA health education of doctors in existing medical curricula contributes to these outcomes. We sought to explore medical students’ experiences of content coverage and mode of delivery, as well as their preparedness, attitudes and learning needs regarding LGBTQIA health education in Australia.

**Methods:**

Using a conceptual framework specific to curricular development, we adapted a previous cross-sectional national survey. This included 28 questions (analysed statistically) and 5 free text responses (analysed deductively using Braun and Clarke’s thematic analysis framework). Data was compared between LGBTQIA and non-LGBTQIA respondents, and clinical and preclinical students.

**Results:**

There were 913 participants from 21 of 23 medical schools, with most preclinical (55%) and clinical (89%) students reporting no teaching specific to LGBTQIA health. Reported content coverage was highest for sexual history taking (30%), and especially low for transgender and intersex health (< 16%), and intersectional LGBTQIA health (< 7%). Participants had positive attitudes towards LGBTQIA health, with 89% agreeing LGBTQIA topics were important and need to be covered in detail. Students desired longitudinal integration of LGBTQIA content, and LGBTQIA community involvement and case-based teaching that allows for interaction and questions. Self-perceived competency was low in all LGBTQIA health topics, although LGBTQIA participants reported higher preparedness than non-LGBTQIA participants.

**Conclusions:**

Majority of survey participants reported limited teaching of LGBTQIA health-specific content, highlighting the limited coverage of LGBTQIA health in Australian medical schools. Participants expressed positive attitudes towards LGBTQIA content and broadly agreed with statements supporting increased integration of LGBTQIA health content within medical curricula.

## Introduction

There is an increasing interest in lesbian, gay, bisexual, transgender, queer, intersex and asexual (LGBTQIA) health content in medical curricula. This interest grows alongside recognition of the health disparities faced by LGBTQIA individuals across mental and general health outcomes, particularly for trans and gender diverse (TGD) people, bisexual people, and those with intersex variations [1–6]. These health disparities can be compounded by a lack of inclusive practices from health care providers [1, 6–8].

International studies on lesbian, gay, bisexual, transgender, queer, intersex and asexual (LGBTQIA) health content in medical curricula suggest there is insufficient teaching on the topic, and little focus given to students’ experiences of the teaching provided [9–13]. Medical curriculum studies from Canada and the United States establish relatively little time dedicated to lesbian, gay, bisexual, transgender (LGBT) health, with a median of 5 h in undergraduate programs [10]. A study in Japan report a median of 1 and 0 h in preclinical and clinical training respectively [13]. Similarly, Australian medical students and doctors report insufficient training on LGBTQIA health issues and inclusive practices, with notable gaps in transgender and intersex health education [9, 14]. A 2017 survey of medical school curriculum administrators in Australia and New Zealand indicated most medical schools (60%) dedicated 0–5 h to LGBTQIA health in preclinical years. Most of this content focused on same-sex sexual activity (80%), with half of the respondents (47%) unsure whether trans and gender diversity was covered in their curricula [9]. Students' experiences of teaching point towards low levels of self-reported preparedness, although their preferences and learning needs have not yet been comprehensively explored, and the samples surveyed were limited (low numbers of students or medical schools) [11, 12]. Overall, the scope, coverage, and assessment of LGBTQIA material was found to be highly variable, with minimal focus given to the engagement and learning needs of students [9]. Given the variability of content, different modes of delivery, and knowledge gaps about student perceptions and needs, a student-focussed approach to the educational deficits in this area are needed.

This study aimed to address the following questions:

1. What are medical students’ recalled experiences of content coverage and mode of delivery of LGBTQIA health education in Australia?
2. What are medical students’ preparedness, attitudes and learning needs in relation to LGBTQIA health education in Australia?

In contrast to an objective audit of content included in LGBTQIA medical curricula (which may or may not align with students’ learning), we sought to go beyond this by taking medical students’ direct perspectives on these matters. Our findings could help to guide and standardise LGBTQIA medical curricular development to meet the learning experiences and needs of students. We envision this research to be of use to medical faculties, institutions, students and advocates in improving LGBTQIA health curricula.

## Methods

This survey was adapted from a previous Medical Deans Australia and New Zealand (MDANZ) survey with permission from the lead author, [9] and this project was approved by the Human Ethics Advisory Group at The University of Melbourne (Project ID 2057068.1). The MDANZ survey instrument was developed from a previous study undertaken in North American medical schools, [10] and adapted to the Australian context. The original survey instrument was validated by a panel of LGBT health experts, and 13 Deans of medical schools [9, 10]. Our adaptation of the survey was further piloted with 20 LGBTQIA medical students to assess the accessibility, clarity, and relevance of the instrument. Where appropriate, questions were subsequently reworded to ensure consistently between their intended meaning and trial participants’ interpretation of the questions.

Though not exhaustive of all topics pertaining to LGBTQIA curricula, the survey instrument is representative of critical topics and priorities in the field of LGBTQIA health. These topics and priorities are represented in the constructs the survey is intended to measure (content and coverage, learning preferences, attitudes and preparedness). As both the original survey instrument and the MDANZ survey were oriented to educators, [9, 10] the wording and content of questions was adapted to better address the student population and elicit their perspectives and experiences.

The Qualtrics survey platform was used to conduct the survey. The survey consisted of 28 questions, comprised of multiple choice, checkbox, Likert scale and numerical answers. An additional 5 free-text questions were included to capture qualitative responses. Participants were not supplied with any additional information relating to survey content prior to completion. A curriculum development perspective was adopted from conceptualisation to analysis, using Kern et al.'s [15] six step conceptual framework, applying steps one to four as below:

1. Problem identification and general needs assessment
2. Targeted needs assessment
3. Goals and objectives
4. Educational strategies
5. Implementation
6. Evaluation

A curricula development framework provides a suitable conceptual framework for this study, as it provides a stepwise progression through which this research can be implemented for the betterment of medical curricula. This framework allows for the research to be implemented as a tool for these groups. In applying this conceptual framework, our problem was identified as a lack of safe and informed medical training on LGBTQIA health, as an upstream contributor to health inequities. This identified problem was based upon a trend in the literature that shows a dearth of LGBTQIA content, co-design and community-led practice in medical curricula globally. We assessed medical student needs through a mixed-methods survey as described below, and subsequently derived goals, objectives, and educational strategies at the meeting point of data and external literature.

These forward-looking steps in curricula development were derived by identifying from the data what elements of comprehensive LGBTQIA health education are lacking and the learning preferences of students in relation to improving their preparedness. We subsequently integrated evidence from the literature to validate the themes that were identified, and the success of the educational strategies recommended in improving LGBTQIA health provision and students experience across various medical education contexts.

Following from Greene et al.’s [16] five categories of purpose for mixed methods research, the use of a mixed-methods approach aimed to develop, complement, initiate, triangulate with and expand on the quantitative data collected. Through providing a space for students to elaborate on their perspectives and experiences, a richer, wider, and more granular understanding of LGBTQIA health coverage was derived from the data. Our mixed methods approach allowed us to explore both the thematic connections across curricula, whilst preserving the unique encounters of students with curricula across the continent. Further, the incorporation of qualitative data in this design approach allowed a grounding in the conceptual and experiential vocabulary of the participants. This grounding in student voices was supportive to our research aim, in particular research question 2, and the overarching focus of this study on eliciting student voices. The processes of implementation and evaluation are to follow (i.e. beyond the immediate scope of this manuscript).

### Participants

Survey participants were students recruited from all 23 medical schools in Australia. Survey invitations were sent to universities’ administrative staff and distributed across newsletters and online learning portals. Additionally, student representatives circulated survey invitations amongst social media affiliated with tertiary institutions and the Australian Medical Student Association. Participants provided informed consent prior to survey response. The survey was active between 9th August and 4th December 2020. From the total 17,884 medical students in Australia, it was not known how many received survey invites via the numerous means described, and therefore the response rate was unknown.

Demographic variables collected were limited as to maximise anonymity, and focussed on intersectional identity markers (which could affect students’ learning experiences, given the complex interplay between LGBTQIA health and other minority populations) and status as a medical student (which could affect the amount of curricular exposure to LGBTQIA health teaching received thus far). Collected demographic variables included participants’ gender; sexuality; intersex status; Aboriginal and Torres Strait Islander status; domestic or international enrolment; metropolitan or rural, regional or remote clinical placement; and progression through degree (e.g. pre-clinical, or final year). The format of intersectional variables was multiple-choice (tick all that apply, with the option of free-text responses). The format of degree progression variables was binary, except for progression through degree which was assessed by one binary variable (pre-clinical or clinical) and two numerical responses (year of study, and total length of degree).

### Data management

Prior to analysis, data was deidentified by removing email addresses. Survey responses were exported into Microsoft Excel format and stored on a secure, password-protected university drive. 17,884 medical students were enrolled in Australia’s 23 medical schools in 2020 [17]. We received responses from 1,016 students (5.7% of national total). Of the total 1,016 responses, 102 were discarded due to survey non-completion, or nonsensical answers (i.e. ticking all options). The cleaned dataset was imported into R for Windows version 4.0.2 (with tidyverse packages) for analysis.

### Data analysis

Data was summarised as mean and standard deviation (for continuous variables with a normal distribution), median and interquartile range [IQR] (for continuous variables with a non-normal distribution), or number and percentage (for categorical variables). The number of missing answers for a particular question was noted, with missing answers excluded from all calculations. Likert scales ranged from 1 to 5, which for the purposes of analysis were simplified into ‘agree’ (4 or 5), ‘neither agree nor disagree’ [3], and ‘disagree’ (1 or 2).

Data was compared between the following demographics: LGBTQIA and non-LGBTQIA respondents, defined as those reporting a heterosexual, non-intersex male/female identity; and final-year students and non-final-year students, who may not yet have been exposed to all educational material throughout medical school. To compare responses between these subgroups, the Mann–Whitney U (Wilcoxon rank-sum) test was used for continuous variables, and the Chi-square test was used for categorical variables, with *p* < 0.05 indicating statistical significance.

Free-text comments were analysed through Braun and Clarke's framework for thematic analysis, utilising a deductive approach with an experiential orientation as an underlying theoretical assumption. Retaining our focus on students’ perception and attitudes, this approach orients analysis toward participants’ experience, internal or relational state regarding phenomena [18]. Braun and Clarke’s six step approach for thematic analysis (as follows) guided the process of analysis in a non-linear manner:

1. Familiarisation with the data
2. Coding the data
3. Generating initial themes
4. Reviewing and developing themes
5. Refining, defining and naming themes
6. Producing the report.

A team of five coders analysed each question using Nvivo and met regularly for discussion, consolidation and collaborative reiteration of themes and codes. Responses were initially analysed separately for first-order codes, then organised into an overarching framework of second-order basic themes and third-order organising themes that were identified from both the qualitative data, and reflective of the quantitative data. The organisation of themes and codes occurred non-linearly, responsive to the recursive process of thematic analysis. In total, 2,327 comments were received and 656 included in the final analysis. Sufficient conceptual depth was determined when no new codes were identified from the data set, as agreed upon by all coders [18]. Unanswered prompts, responses without content (e.g. “nil”), and nonsensical responses were discarded for analysis.

The reiterative grouping of codes and generation of themes was guided by our research questions. For example, the topic of self-reported learning needs focussed coding on the learning experience of participants. Frequently occurring codes pertaining to learning needs (such as ‘clinical experience’, ‘longitudinal inclusion’ or ‘interactive learning’) were refined over the coding period, then grouped under basic themes according to the different dimensions of learning experience they represented themes (‘clinical practice’, ‘content’ or ‘format’). These themes were generated and named through a process of interrogating, and reinterrogating, the conceptual congruency of the codes. Organising themes were then created to identify the conceptual ties between themes and codes (‘curricula development’), with the intention of addressing our research foci.

Both semantic and latent coding were employed, as was appropriate to the relationship between the qualitative data and quantitative data [18]. Semantic coding was utilised when triangulating between data sets. For example, codes under the basic theme ‘format’ organise any description of learning format reported by participants, and were applied (where appropriate) to substantiate the desired formats present under ‘Learning Preferences’ (Table 5 in the quantitative data). Latent coding was of greater use in developing and expanding on patterns present in the quantitative data. For example, the codes constituting the 'Educational Environment' organising theme categorise responses that specifically address the diverse experiences and perspectives of both LGBTQIA and non-LGBTQIA students. This coding practice allowed us to better interpret and compare the disparities in quantitative data on metrics like preparedness and attitudes between these two groups.

### Reflexivity

The research team interpreting the data was comprised of members of diverse positionalities from the queer and gender diverse community. We occupy different cultural standpoints, and work within the medical field as either medical students, public health professional and/or practicing doctors. Our embeddedness within both the LGBTQIASB + (lesbian, gay, bisexual, trans, queer, intersex, asexual, sistergirl and brotherboy) community and the medical field may have engendered a shared interpretation of the data and coding practice. Given the demographics of our participants (medical students with a 45.9% response rate from LGBTQIA peoples), we could thus be understood to have taken an insider position. Our relationship to LGBTQIA health and collective orientation to improving healthcare for this group undeniably influenced our interpretation of data, and acted as an organising concept through which themes were generated. Remaining mutually cognisant of this relationship, encouraging one another to interrogate the biases this may produce, exploring multiple perspectives and through this establishing a recursive and reiterative process was central to our coding practice.

In alignment with Beals et al. [19] we problematise this idea of insider and outsider positionality in LGBTQIA research, as all researchers bring situated knowledge to a coding process that are neither entirely subjective nor objective. Given the intersectional and heterogenous nature of identity in the queer, intersex and gender diverse community, and the variance in medical student participant demographics, it is impossible to fully occupy an insider position. Following from this, the personal, cultural and professional diversity of the research team worked to minimise shared bias, while also provide access to different experiences and prior knowledges that assisted the generation of relevant themes. As a team, we engaged with the subjective/objective tension that bridges insider/outsider epistemologies and utilised the multiplicity of our perspectives in a collaborative (rather than consensus) coding practice.

## Results

### Demographics

*N* = 913 students completed the survey, with 359 (40.4%) recruited through social media and 299 (32.7%) recruited through university channels (newsletters, portals etc.). A definitive LGBTQIA identity was expressed by 419 (45.9%), whilst 406 (44.5%) were non-LGBTQIA (cisgender, heterosexual, and non-intersex). Regarding gender identity, 298 (33.1%) identified as male, 577 (64.2%) as female, and 34 (3.8%) as trans and gender diverse. Of 838 reporting ethnicity, the most common were 426 (50.8%) Australian European, 182 (21.7%) South-East Asian, and 67 (8.0%) North-East Asian. Broader participant demographics are outlined in Table 1.

**Table 1:** Participant demographics

LGBTQIA	N (%)	Medical school enrolments 2020
LGBTQIA	419 (45.9%)	-
Non-LGBTQIA^a^	406 (44.5%)	-
**Gender**		
Female	577 (64.2%)	9353 (52.3%)
Male	298 (33.1%)	8513 (47.6%)
Queer	86 (9.6%)	-
Trans and gender diverse	34 (3.8%)	-
Questioning/unsure	14 (1.6%)	-
**Sexuality**		
Heterosexual	453 (51.4%)	-
Bisexual/Pansexual	270 (30.6%)	-
Queer	119 (13.5%)	-
Gay	110 (12.5%)	-
Lesbian	55 (6.2%)	-
Questioning/unsure	46 (5.2%)	-
Asexual/Aromantic	70 (7.9%)	-
**Intersex**		
Intersex	4 (0.5%)	-
**Aboriginal and Torres Strait Islander**		
Aboriginal and/or Torres Strait Islander	20 (2.5%)	403 (2.3%)
**Medical student status**		
Domestic	775 (87.9%)	14,933 (83.5%)
International	107 (12.1%)	2951 (16.5%)
Location of schooling		
Metropolitan	739 (83.9%)	12,590 (70.4%)
Rural, Regional or Remote	142 (16.1%)	5294 (29.6%)
Stage of schooling		
Preclinical	459 (51.2%)	-
Clinical	427 (48.8%)	-
Final year	153 (17.4%)	3656 (20.1%)
Non-final year	725 (86.2%)	-

^a^Defined as those reporting a heterosexual, non-intersex male/female identity only; includes those who were ‘questioning’ their identity who otherwise fit this definition

### Attitudes towards LGBTQIA patients

Students largely reported positive attitudes towards LGBTQIA patients, with no statistically significant differences between LGBTQIA and non-LGBTQIA participants, or between final-year and non-final-year students (data not shown). Student attitudes are displayed in Table 2.

**Table 2:** Student attitudes towards LGBTQIA health

		**LGBTQIA vs non-LGBTQIA**		
**Statement**	**Agree, N (%)**	**LGBTQIA, N (%)**	**Non-LGBTQIA, N (%)**	***p*-value**
I feel comfortable taking a sexual history from gay, lesbian and bisexual patients	566 (83.2%)	306 (91.3%)	213 (73.2%)	**χ**^**2**^**(1) = 36.2, *p* < 0.001**
Same-sex attraction is a natural expression of human sexuality	657 (92.2%)	326 (97.6%)	260 (89.0%)	**χ**^**2**^**(1) = 19.1, *p* < 0.001**
As a future doctor, I would feel comfortable providing treatment for gender affirmation to trans and gender diverse patients^a^	504 (74.1%)	278 (83.2%)	192 (65.7%)	**χ**^**2**^**(1) = 25.4, *p* < 0.001**
Trans and gender diverse identities are reversible with psychiatric intervention	24 (3.5%)	5 (1.5%)	16 (5.5%)	**χ**^**2**^**(1) = 7.7, *p* = 0.006**
Genital atypia related to intersex variations should be corrected at birth to align with either a male or female genitalia	54 (7.9%)	13 (3.9%)	35 (12.0%)	**χ**^**2**^**(1) = 14.4, *p* < 0.001**
I feel comfortable in providing care to people born with intersex variations^b^	434 (63.8%)	229 (68.4%)	174 (59.6%)	**χ**^**2**^**(1) = 5.2, *p* = 0.022**
People who identify as asexual require medical assessment and intervention	25 (3.7%)	16 (2.9%)	9 (7.1%)	**χ**^**2**^**(1) = 5.3, *p* = 0.022**
LGBTQIA topics are important and need to be covered in detail at medical school	649 (89.2%)	338 (94.2%)	265 (85.2%)	**χ**^**2**^**(1) = 14.8, *p* < 0.001**
The healthcare needs of First Nations LGBTQIA people are important and need to be covered in detail at medical school	639 (88.1%)	332 (92.5%)	263 (85.1%)	**χ**^**2**^**(1) = 9.3, *p* = 0.002**
At present, there is sufficient coverage of LGBTQIA topics at medical school	107 (14.7%)	39 (10.9%)	56 (18.0%)	**χ**^**2**^**(1) = 6.9, *p* = 0.009**
I feel confident that the other students in my cohort are well prepared to give medical care to LGBTQIA people	100 (13.7%)	39 (10.9%)	50 (16.1%)	**χ**^**2**^**(1) = 3.9, *p* = 0.047**

Except for items noted below, there were no statistically significant differences between final year and non-final year students

^a^Final years vs non-final years: 84 (63.6%) vs 418 (76.6%), χ2(1) = 9.2, *p* = 0.002

^b^Final years vs non-final years: 75 (56.4%) vs 359 (65.9%), χ2(1) = 4.2, *p* = 0.041

### Attitudes towards LGBTQIA health in medical education

Student attitudes and perceptions towards LGBTQIA health in medical curricula were largely positive. Participants who identified as LGBTQIA reported higher positive attitudes towards LGBTQIA health compared to non-LGBTQIA participants across all statements. Student attitudes towards LGBTQIA health are displayed in Table 2.

### Preclinical and clinical teaching

**Fig. 1:**
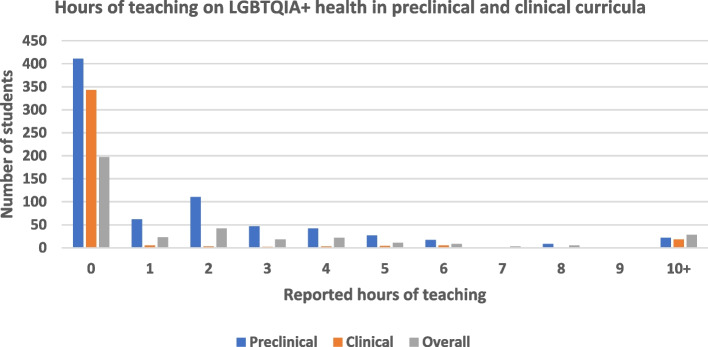
Hours of preclinical and clinical LGBTQIA teaching reported by students. The overall category includes participants with data on both preclinical and clinical teaching

Regarding clinical teaching, 43 (10.4%) students had completed clinical rotations specific to LGBTQIA health; of those that had, the median duration was six hours. Overall, amongst the 358 participants reporting data on both preclinical and clinical teaching, 197 (55.0%) reported no teaching at all, with the mean number of hours of overall teaching being 2.2 h (Fig. 1). Some respondents were ‘Unsure’ about the hours of preclinical (*n* = 147, 17.0%) and clinical (*n* = 29, 7.0%) teaching they had received.

There was notable inter-university variability. The median percentage of respondents reporting any content coverage was 60.0% [44.2%, 66.7%] for any preclinical teaching. There was greater variability in clinical teaching, with a median of respondents reporting any content being 9.4% [0.8%, 30.0%].

### Preparedness and content coverage

The reported content coverage of LGBTQIA health competencies is shown in Table 3. Reported content coverage did not significantly differ between final year and non-final year respondents.

**Table 3:** Content coverage of LGBTQIA health competencies

Competency	Content coverage, N (%)
Take a comprehensive sexual history from lesbian, gay or bisexual patients	191 (29.8%)
Use terms and definitions in the LGBTQIA acronym	148 (22.5%)
Take a history of presenting complaint for a common medical condition from an LGBTQIA patient	144 (21.9%)
Take a comprehensive sexual history from trans or gender diverse patients	100 (15.6%)
Perform a common physical exam (i.e. cardiovascular exam) on an LGBTQIA patient	95 (14.5%)
Discuss alcohol, tobacco or other drug use with LGBTQIA patients	93 (14.2%)
Address the mental health needs of lesbian, gay, bisexual people	73 (11.4%)
Refer LGBTIQ patients to community organisations and resources for support	67 (10.2%)
Address the mental health needs of transgender and gender diverse people	50 (7.8%)
Provide culturally safe care to First Nations LGBTQIA people (i.e. Takatāpui, Sistergirl, Brotherboy)	42 (6.4%)
Discuss intimate partner violence with LGBTQIA patients	41 (6.3%)
Discuss options for hormone replacement therapy with trans and gender diverse patients	33 (5.1%)
Address the mental health needs of intersex individuals	31 (5.0%)
Address the healthcare needs of individuals born with intersex variations	31 (4.8%)
Address healthcare needs of LGBTQIA people of colour	26 (4.0%)
Address healthcare needs of LGBTQIA migrants, refugees and asylum seekers	26 (4.0%)
Address the healthcare needs of LGBTQIA people living with a disability	24 (3.7%)
Discuss options for gender affirmation surgery with trans and gender diverse individuals	22 (3.4%)

Self-reported preparedness in LGBTQIA health competencies is shown in Table 4. For all competencies, students reported preparedness at greater rates than content coverage.

**Table 4:** Self-reported preparedness of LGBTQIA health competencies

		**LGBTQIA vs non-LGBTQIA**			**Final year vs non-final year**		
**Competency**	**Prepared, N (%)**	**LGBTQIA, N (%)**	**Non-LGBTQIA, N (%)**	**Difference between groups**	**Final year, N (%)**	**Non-final year, N (%)**	**Difference between groups**
Use terms and definitions in the LGBTQIA acronym	520 (79.1%)	294 (91.0%)	188 (66.7%)	**χ**^**2**^**(1) = 55.1, *p* < 0.001**	106 (81.5%)	413 (78.7%)	χ^2^(1) = 0.5, *p* = 0.470
Take a history of presenting complaint for a common medical condition from an LGBTQIA patient	510 (77.5%)	266 (82.1%)	203 (72.0%)	**χ**^**2**^**(1) = 8.8, *p* = 0.003**	114 (87.7%)	394 (74.9%)	**χ**^**2**^**(1) = 9.8, *p* = 0.002**
Perform a common physical exam (i.e. cardiovascular exam) on an LGBTQIA patient	541 (82.3%)	285 (88.0%)	220 (78.0%)	**χ**^**2**^**(1) = 10.7, *p* = 0.001**	123 (94.6%)	416 (79.2%)	**χ**^**2**^**(1) = 16.9, *p* < 0.001**
Provide culturally safe care to First Nations LGBTQIA people (i.e. Takatāpui, Sistergirl, Brotherboy)	174 (26.5%)	84 (25.9%)	76 (27.0%)	χ^2^(1) = 0.1, *p* = 0.775	47 (36.2%)	125 (23.8%)	**χ**^**2**^**(1) = 8.2, *p* = 0.004**
Refer LGBTIQ patients to community organisations and resources for support	283 (43.1%)	158 (48.8%)	106 (37.6%)	**χ**^**2**^**(1) = 7.7, *p* = 0.006**	59 (45.4%)	222 (42.3%)	χ^2^(1) = 0.4, p = 0.523
Address healthcare needs of LGBTQIA people of colour	292 (44.5%)	152 (47.1%)	122 (43.3%)	χ^2^(1) = 0.9, *p* = 0.349	67 (51.5%)	223 (42.6%)	χ^2^(1) = 3.4, *p* = 0.065
Address healthcare needs of LGBTQIA migrants, refugees and asylum seekers	232 (35.4%)	110 (34.0%)	105 (37.4%)	χ^2^(1) = 0.8, *p* = 0.381	57 (44.2%)	173 (33.0%)	**χ**^**2**^**(1) = 5.7, *p* = 0.017**
Address the healthcare needs of LGBTQIA people living with a disability	233 (35.5%)	115 (35.5%)	104 (36.9%)	χ^2^(1) = 0.1, *p* = 0.723	58 (44.6%)	173 (33.0%)	**χ**^**2**^**(1) = 6.2, *p* = 0.013**
Discuss alcohol, tobacco or other drug use with LGBTQIA patients	473 (72.2%)	238 (73.7%)	202 (71.6%)	χ^2^(1) = 0.3, *p* = 0.572	112 (86.2%)	359 (68.6%)	**χ**^**2**^**(1) = 15.9, *p* < 0.001**
Discuss intimate partner violence with LGBTQIA patients	288 (43.9%)	153 (47.4%)	111 (39.4%)	**χ**^**2**^**(1) = 3.9, *p* = 0.048**	74 (56.9%)	212 (40.5%)	**χ**^**2**^**(1) = 11.5, *p* < 0.001**
Take a comprehensive sexual history from lesbian, gay or bisexual patients	484 (75.5%)	256 (80.5%)	193 (71.0%)	**χ**^**2**^**(1) = 7.3, *p* = 0.007**	118 (91.5%)	364 (71.4%)	**χ**^**2**^**(1) = 22.4, *p* < 0.001**
Take a comprehensive sexual history from trans or gender diverse patients	365 (56.9%)	188 (59.1%)	149 (54.8%)	χ^2^(1) = 1.1, *p* = 0.288	90 (69.8%)	273 (53.4%)	**χ**^**2**^**(1) = 11.2, *p* < 0.001**
Discuss options for hormone replacement therapy with trans and gender diverse patients	100 (15.6%)	56 (17.6%)	38 (14.0%)	χ^2^(1) = 1.4, *p* = 0.229	28 (21.7%)	71 (13.9%)	**χ**^**2**^**(1) = 4.8, *p* = 0.029**
Discuss options for gender affirmation surgery with trans and gender diverse individuals	86 (13.4%)	49 (15.5%)	30 (11.0%)	χ^2^(1) = 2.5, *p* = 0.116	19 (14.7%)	66 (12.9%)	χ^2^(1) = 0.3, *p* = 0.593
Address the healthcare needs of individuals born with intersex variations	98 (15.3%)	44 (13.8%)	45 (16.6%)	χ^2^(1) = 0.8, *p* = 0.360	23 (17.8%)	74 (14.5%)	χ^2^(1) = 0.9, *p* = 0.348
Address the mental health needs of lesbian, gay, bisexual people	365 (56.9%)	208 (65.4%)	130 (48.0%)	**χ**^**2**^**(1) = 18.2, *p* < 0.001**	94 (72.9%)	269 (52.7)	**χ**^**2**^**(1) = 17.0, *p* < 0.001**
Address the mental health needs of transgender and gender diverse people	288 (44.9%)	156 (49.1%)	112 (41.2%)	χ^2^(1) = 3.7, *p* = 0.055	75 (58.1%)	211 (41.3%)	**χ**^**2**^**(1) = 11.8, p < 0.001**
Address the mental health needs of intersex individuals	229 (35.7%)	119 (37.4%)	98 (36.1%)	χ^2^(1) = 0.1, *p* = 0.752	61 (47.3%)	166 (32.5%)	**χ**^**2**^**(1) = 9.8, *p* = 0.002**

### Learning preferences

Students overwhelmingly preferred learning experiences facilitated by member of the community to be the most effective learning modalities. Learning preferences did not differ for between LGBTQIA and non-LGBTQIA participants, or between final-year and non-final-year students (Table 5).

**Table 5:** Learning preferences reported by students

Learning preferences	N (%)
Lectures delivered by members of the LGBTQIA community	704 (86.4%)
Small workshops or tutorials facilitated by members of the LGBTQIA community	552 (67.7%)
Sharing of lived experience by members of the LGBTQIA community	462 (56.7%)
Panel discussions with both clinicians and LGBTQIA community members	460 (56.4%)
Clinical placements focused on LGBTQIA health	448 (55.0%)
Clinical case discussions	351 (43.1%)
Lectures delivered by faculty members/clinicians	244 (29.9%)
Video scenarios demonstrating best practice	240 (29.4%)
Small workshops or tutorials facilitated by faculty members	552 (26.6%)
Online modules	179 (22.0%)
Conferences focused on LGBTQIA health	157 (19.3%)
Content delivered by other medical students	114 (14.0%)
Reflective practice (i.e. journals or reflective essays)	89 (10.9%)

### Assessment

Only 395 (48.2%) of students reported assessment of LGBTQIA health, and 424 (51.8%) of students reported no assessment of LGBTQIA health. Of those reporting assessment, the most common formats were questions in written exams (*n* = 167, 42.3%), short-case clinical discussions (*n* = 52, 13.2%), Objective Structured Clinical Examinations (OSCEs) (*n* = 46, 11.6%), and evaluation by standardised patient actors (*n* = 42, 10.6%).

### Qualitative results

**Fig. 2:**
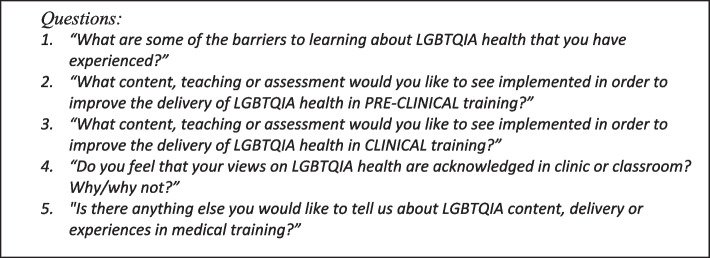
Qualitative free text box questions

**Fig. 3:**
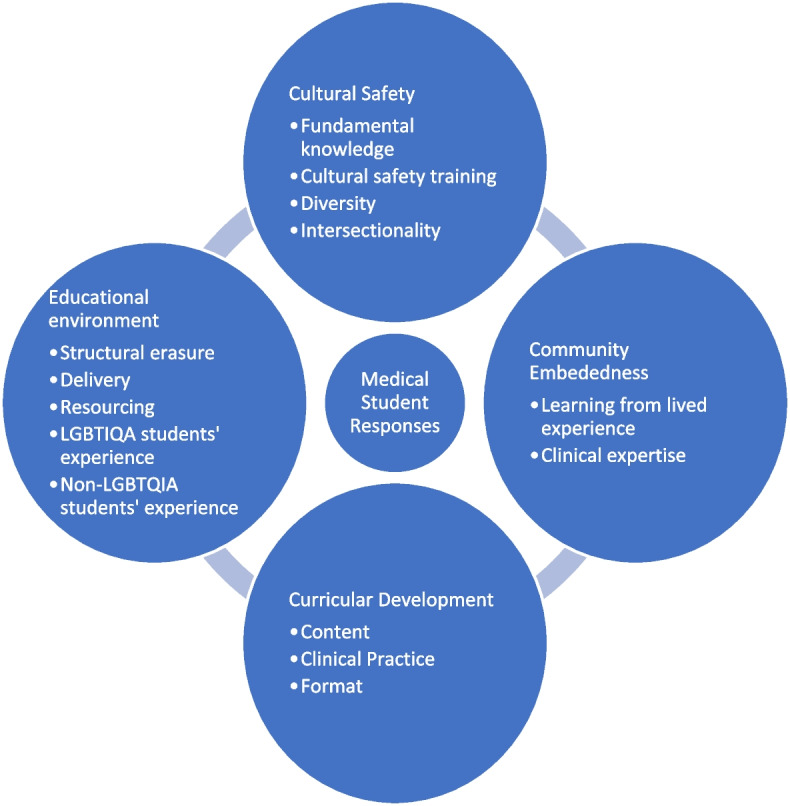
Key themes in the qualitative data

Qualitive data analysis of free-text responses (Fig. 2) identified four key themes (Fig. 3).

### Theme 1: Cultural safety

Participants articulated environments and relationships in which peoples feel safe or unsafe within their identity, culture and community group, characterised by an absence or presence of discrimination. This focus was identified in text responses related to curricula content, skill development, and institutional experiences. The organising theme ‘Cultural Safety’ was generated as representative of the conceptual alignment between the basic themes 'fundamental knowledge', 'cultural safety training', 'diversity' and 'intersectionality'. These were understood as elements of culturally safe learning environments, relationships, and practice.

Learning preferences for specialised training such as cultural safety and communication skills workshops were frequently repeated throughout the codes in text. This preference develops upon the quantitative discrepancies in self-reported preparedness to provide culturally safe care to diverse LGBTQIA patients, and desires for improved curricula delivery and content.

*“We need workshops on cultural competency and on competency in delivering safe care to LGBTQIA people,” (R_vx)*

*"[My university] did have some LGBTQIA simulated patients in our communication skills course. One of these was to take a sexual history from a gay male patient and included asking about specific sexual practices. They also had a station with a transgender SP who was actually played by a transgender person. I really appreciated this!” (R_Rb)*

### Theme 2: Community embeddedness

Responses addressed the centring of LGBTQIA expertise and lived experience in the creation and teaching of LGBTQIA medical curricula. Codes pertaining to 'learning from lived experience' and 'clinical expertise' were linked through the varied but related ways in which they describe the lack or presence of, or desire for, community embedded curricula and delivery.

Preferences for LGBTQIA curricula to be embedded in the LGBTQIA community was present throughout the free-text responses. This was informed by a professed lack of guidance by LGBTQIA community members, educators and health experts, a lack of practical rotation through LGBTQIA health spaces and a perception that curricula design was not led by LGBTQIA peoples.

*“Teaching FROM members of the LGBTQIA community” (R_3r )*

*“Our classes on this topic [LGBTQIA health] were delivered by cisgender heterosexual doctors who often did not understand these issues in depth.” (R_31)*

The dearth of community embeddedness was associated with an absence of authenticity, intersectionality, and representation. This perception was in relation to reports of unchallenged practices such as stereotyping and misgendering. Aligning with student preferences for community embedded teaching elicited from the quantitative data, this theme expands upon why students hold these preferences, and clarifies this imperative through a grounding in student experience.

*“I've raised the issue of misgendering to a consultant before and they were dismissive, arrogant and continued to misgender the patient in front of them for the entire admission.” (R_0B)*

### Theme 3: Curricular representation

Responses referred to the integration (or lack) of LGBTQIA peoples, communities, and healthcare needs in medical curricula. This organising theme was generated as representative of the conceptual connections between the basic themes 'content', 'clinical practice' and 'format' as essential elements of medical curricula, and the elements of curricula most frequently explored in the qualitative data.

Codes pertaining to diversity and representation were continuously identified throughout the text responses. The predominance of cis-gendered, heterosexual teaching staff on medical faculties was repeatedly raised as an issue:

*“All teaching is based on heteronormative framework and there is a constant queer erasure in medical curriculum content delivery” (R_Zf)*

Students perceived heightened levels of discomfort in discussing LGBTQIA health, poor staff preparedness, little reflexivity, outdated knowledge, discriminatory behaviours and the silencing of LGBTQIA community members. Multiple codes referred to faculty cultures of othering and erasure, accompanied by insufficient institutional mechanisms to address this culture:

*“[I] do not feel like i can speak up in a clinical setting even when people are being homophobic or misgendering others because I am visibly queer myself and it would turn it back on me.” (R_2r)*

Issues of representation within curricula itself was similarly present, with the codes emphasising a lack of content and/or depth occurring frequently. Further, the LGBTQIA resource supplementation required to fill in gaps in curricula was seen as invalid or simply not occurring.

*“Literally anything would be better than what we're getting now, which is nothing except for a single HIGHLY offensive lecture on people who are intersex.” (R_YP)*

*“Lack of content taught to students, no extra reading/information provided for students who would like to learn more” (R_to)*

*“More casual inclusion of LGBTQIA*+ *scenarios in practical and discussion classes; normalising the experiences of LGBTQIA*+ *patients.” (R_3K)*

These findings complement and triangulate with the lack of content reported in the quantitative data, develop insight into preferences for lived experience teaching, and expand upon the low rates of self-reported preparedness in LGBTQIA health competencies, particularly regarding LGBTQIA peoples with intersectional identities.

### Theme 4: Educational environment

Participants explained the context, and experience of context, in which medical curricula is being delivered. This organising theme was interpreted as the conceptual connection between the basic themes: 'structural erasure', 'delivery', 'resourcing', 'LGBTIQA students' experience' and 'non-LGBTQIA students' experience' due to their representation of educational context beyond and within curricula.

Students described a deficit of safe facilitation by faculty between students, as well as between the student body and the LGBTQIA community. Both LGBTQIA students and non-LGBTQIA students expressed feeling unsafe in their classrooms. This experience of feeling unsafe took multiple forms. The recurrent issues for queer students related to a paradoxical sense of hypervisibility and invisibility (for example being called upon as an expert for LGBTQIA issues, whilst having your queerness and/or gender diversity suppressed elsewhere) and the emotional toll of having to navigate discriminatory environments:

*“Personally [I experience] anxiety about facing prejudiced views from teachers/students.” (R_1g)*

*“Where I have been able to contribute, my opinions and views have been acknowledged positively by both classmates and facilitators of any sessions…. it can be difficult sharing an experience as the only LGBTQIA*+ *identifying student in the room.” (R_3k)*

*“I keep pretty quiet about LBTQIA health unless I’m in small groups, and even then there is a lot of emotional labor involved. I have been surprised by the amount of homophobic and transphobic jokes from medical students and lecturers alike.” (R_3M)*

For non-LGBTQIA students, a fear of offending LGBTQIA peoples due to a lack of knowledge (for example around the correct use of terminology) and a sense of feeling unheard was identified in data such as the following:

*“If I have a differing viewpoint about LGBTQI health and I want to discuss it with an LGBTQI person, or expert in the area, to get a better understanding for myself, they make me feel bad about the way I think and it is difficult to learn more. (R_30)*

*“[I’m] worried about offending someone or saying the wrong thing” (R_1f)*

*“[A]s a person with a very boring heteronormative, cis lived experience, I do fear saying or doing the wrong thing and upsetting or traumatising patients identifying in this way... which makes me nervous!” (R_C4)*

This initiates and expands upon the quantitative data displaying low coverage of cultural safety and communication content, and points to a need for better-facilitated classrooms.

## Discussion

This study is the first of its kind in Australia to use student data to map the perceived depth and breadth of LGBTQIA health education in Australian medical curricula. In contrast to previous literature surveying medical school deans providing more administrative data, this study has the benefit of focussing on direct student engagement and specific learner needs [9, 10, 13]. Through evidencing specific gaps and drawing on student experience, this study facilitates curricular recommendations driven by data. Given the limited LGBTQIA health education in Australian medical curricula and worldwide, alongside evidenced poorer health outcomes and healthcare experiences for this group globally, it is imperative that we continue developing LGBTQIA health teaching in medical curricula. [1, 2, 6–9, 20–22].

We found that although students regarded LGBTQIA health education as important, relevant teaching was lacking, with most students not feeling confident that their peers were well prepared to provide care to LGBTQIA individuals. Consistent with a 2017 survey of medical school curriculum administrators in Australia and New Zealand, reported teaching hours were low in both clinical and preclinical years [9]. Topics related to intersex health, TGD health, and the health of individuals with intersecting identities (including First Nations LGBTQIA people) were found to have the lowest reported coverage, alongside consistently low self-rated preparedness and confidence. We identified community involvement, increased content, and longitudinal integration into the curriculum as important considerations for the delivery of LGBTQIA health in medical teaching. These findings can inform and guide ongoing curricular development, whilst highlighting opportunities for further medical education research.

### Content coverage

Consistent with previous studies in Australia and internationally, reported content coverage and assessment regarding LGBTQIA health was generally low [9, 10, 14, 23, 24]. Only a third of our participants reported content in their preclinical curriculum, and only a tenth reported exposure to clinical rotations specific to LGBTQIA health. The percentage of students reporting any LGBTQIA health teaching was highly variable between universities, with some universities having no students reporting teaching, to others having all students reporting teaching.

Where present, content related to LGBTQIA health was largely focused on sexual health. The sexual health of gay, lesbian and bisexual patients had the highest coverage for any given topic, with close to a third reporting having covered it in their curriculum. On the other hand, coverage related to the health of intersex, TGD and other underserved communities were reported by less than a tenth of respondents. These findings correlate with negative healthcare experiences of TGD and intersex people reported in Australia and abroad, [25–29] many of whom have limited access to safe and sensitive care, for example, there being an excessive focus on sexual practices when presenting for non-sexual complaints [30–32]. They support the need for improved education of students and medical providers internationally [26, 27, 32].

Despite receiving the same coverage, LGBTQIA students reported higher levels of self-assessed preparedness (Table 4), greater desire for more coverage, and lesser confidence in their peers to provide safe care when compared to non-LGBTQIA students (Table 2). These discrepancies may be attributed to learning gained through lived experiences, greater awareness of deficits, alongside greater engagement in non-institutional and student-led learning. To ascertain, more research needs to be done on this topic.

### Teaching and delivery

Despite low reported coverage, most (89.2%) participants thought LGBTQIA health was important and should be covered in their medical training. However, few participants thought that the current coverage was adequate, or felt confident that their peers would be able to provide adequate care to LGBTQIA patients. This suggests students perceive LGBTQIA health as a priority area but are aware of the deficiencies in their curricula. The qualitative data reflects these findings; students frequently reported dissatisfaction with the quality and quantity of content. Additionally, issues with safe classroom facilitation and teacher capability were repeatedly raised.

In terms of addressing gaps in curricula, participants expressed a strong preference for learning from members of the LGBTQIA community, for example lectures delivered by members of the LGBTQIA community. Other preferred modes of delivery included LGBTQIA-led teaching in small workshops and tutorials, learning about lived experiences, and panel-led discussions with clinicians. Content describing a “lack of learning from lived experiences”, and desired “interactive learning” were predominant in the qualitative data set, further demonstrating the gaps in current teaching practices. In the free-text responses, students called for increased LGBTQIA-led teaching and “case-based teaching” where discussion and direct feedback can occur. Whilst a growing body of evidence suggests that one-off educational interventions may improve attitudes, knowledge and skills in medical students, the educational effects of short-term interventions are often lost to follow-up [33]. Participants further commented that they wanted teaching to be integrated via “longitudinal inclusion” into the curriculum. Reponses speaking to “stigma” and “phobia” in the curricula content, infrastructure and delivery were frequent and reflected students’ desires to move away from a pathologising framework and have LGBTQIA identities normalised in healthcare. We hypothesise that normalising and integrating LGBTQIA patients in medical school may have a preventative effect on the stigma and discrimination that these patients regularly experience in healthcare settings.

There is little data available specifically on the association between increased LGBTQIA curricula content and the improvement of patient outcomes. However, studies that focus on improved outcomes for underserved groups, such as First Nations peoples, demonstrate better clinical outcomes, patient experiences and provider confidence [11, 34, 35]. Some studies also displayed increased understanding thereby efficacy of action related to concepts such as bias, discrimination and advocacy after targeted curricula inclusion [33, 36–39]. Although minority experiences of discrimination are not and should not be commensurable, a common thread of enhanced patient safety can be drawn alongside a recognition of the intersections of identity between these groups.

Informing our hypothesis is the model of medical education as it currently stands, in which high-quality curricular content leads to better provision of care and better patient outcomes. As Ramsden [40] inquires, why wouldn't this same model apply to medical knowledge often relegated to the realm of the socio-political? Prior research internationally has found that greater LGBT patient contact and education hours significantly improved clinical preparedness and knowledge in LGBT healthcare [12]. As a result, medical curricula should consider longitudinal integration to allow medical students further opportunities to cement their knowledge and clinical skills when working with LGBTQIA patients.

Improved content exposure and delivery is particularly needed for intersex and TGD health education. Both qualitative and quantitative analyses demonstrated a clear deficit in both teaching and student preparedness when it comes to intersex and TGD health. This is important as intersex and TGD patients have poor health experiences and outcomes in the Australian health care system, and healthcare systems globally [25, 27, 33]. We suggest that addressing intersex and TGD health in medical education could lead to an improvement in future health outcomes.

### Limitations

This study has several limitations. Firstly, our response rate of 913 students equates to only 5.1% of medical students across Australia [41]. The sample was skewed towards female-identifying individuals (approximately two-thirds of respondents) and LGBTQIA individuals (comprising half the sample). Similarly, non-LGBTQIA respondents may be biased towards those with an interest, knowledge, or experience in LGBTQIA health. Moreover, only respondents who were in their final year could be expected to have had complete exposure to their medical curriculum. To address these limitations, we compared LGBTQIA vs non-LGBTQIA individuals and final-year vs non-final year students in their responses. Since this involved many comparisons, this aspect of our analyses was predisposed to type I error.

Secondly, the psychometric validity of the survey was limited, as only a small number of trial participants were used to evaluate whether questions were interpreted and answered by participants in the manner intended. Questions rated on a Likert scale offered no further instructions or clarifications, leaving open to interpretation certain word choices (e.g. ‘feel’) which may have had heterogeneous interpretations by participants. However, in its original incarnation, the survey instrument was validated with LGBT health experts and medical deans, and we underwent out own piloting with a small number of medical students to assess for accessibility, clarity, and relevance. Reassuringly, the reliability of the survey (and reproducibility of results) was bolstered by a mixed methods approach, triangulating our data and providing opportunity for free text expression alongside fixed quantitative responses.

Thirdly, although we assessed self-reported delivery, coverage, attitudes, and preparedness, we did not assess the degree to which this translates into clinical knowledge, skills or competencies that could be objectively demonstrated. We likewise did not assess general self-rated preparedness to identify whether a lack of preparedness was specific to competency in LGBTQIA health. Moreover, we did not assess the quality of the content being delivered, e.g. whether education delivered was stigmatising and outdated in nature. Hence, we recommend further research and a formal audit into the quality of LGBTQIA medical curricula at present. Longitudinal research into the association between LGBTQIA curricula content and improved patient outcomes is also needed.

Lastly, while a mixed methods approach was employed, a direct relationship between the phrasing of the quantitative survey questions and free text questions would have allowed for greater clarity in triangulating, developing, and expanding the data between methods.

## Conclusion

This large survey of medical students highlights areas of opportunity in Australian medical curricula, and medical curricula globally. Consistent with previous research both in Australia and internationally, reported teaching hours and content coverage of LGBTQIA health were limited, particularly for the health of intersex, TGD, and LGBTQIA peoples with intersecting identities. While students generally reported feeling comfortable providing health care to LGBTQIA patients, they did not feel prepared for many competencies, and did not feel confident that their peers were well prepared to provide care to LGBTQIA patients. Our findings support the need for increased preclinical and clinical content coverage of LGBTQIA health topics through integrated, longitudinal small group teaching and teaching led by LGBTQIA individuals, alongside greater depth and diversity in assessment. These findings can help guide further research in medical education and help inform the ongoing development of LGBTQIA medical curricula.

### Definitions

LGBTQIA: Lesbian, Gay, Bisexual, Transgender, Queer, Intersex and Asexual.

LGBTQIASB + : Lesbian, Gay, Bisexual, Transgender, Queer, Intersex, Asexual, Sistergirl and Brotherboy.

Lesbian: Women or non-binary people who experience sexual or romantic attraction to people of the same gender.

Gay: Men or non-binary people who experience sexual or romantic attraction to people of the same gender.

Bisexual: People who experience sexual or romantic attraction to more than one gender.

Transgender (‘trans’) and Gender Diverse: An umbrella term used to describe all those whose gender identity is different from the sex assigned to them at birth. This includes people who may identify as non-binary, having no gender, fluid gender, multiple genders, a gender other than man or woman, or consider their gender in another paradigm from these.

Queer: An umbrella term for those whose gender or sexual/physical/romantic attraction differs from cis-hetero norms of gender identity and sexuality.

Intersex: A general term describing congenital variations in anatomical, physiological, or genetic sexual characteristics. This includes primary and secondary sexual characteristics which do not fit cis-normative and/or endosex-normative medical conceptions of male or female bodies. While these variations are sometimes clinically labelled as Disorders of Sex Development (DSD) or hermaphroditism, these descriptors are derogatory and inappropriate.

Asexual/ Aromantic: Individuals who experience little or no sexual or romantic attraction to others regardless of gender.

A Note on the Acronym.

At the time the survey was undertaken, ‘trans’ was used as inclusive of all culturally specific trans identities, such as sistergirl and brotherboy, or two spirit. However, the current best practice is to delineate culturally specific trans identities to highlight their unique relationship to cultural protocol and practice, alongside the antecedence of gender diversity to colonialism. It is imperative we acknowledge the queer and gender diverse First Nations people of the land we are writing from. For this reason, the acronym LGBTQIASB + , in which the SB stands for sistergirl and brotherboy, is used when referring to the queer, intersex and gender diverse community as separate from our research focus. LGBTQIA is used when referring to the specific population included within in medical curricula, as these were the parameters used in the survey. Other incarnations of the acronym, such as LGBT or TGD, are similarly used to reflect the scope of research they are derived from, or to reference specific identities within the community.

## Data Availability

The datasets used and/or analysed during the current study are available from the corresponding author on reasonable request.
